# Comparison of ultrasound-guided radiofrequency ablation and hepatectomy for colorectal liver metastasis: A protocol for systematic review and meta-analysis

**DOI:** 10.1097/MD.0000000000032858

**Published:** 2023-02-03

**Authors:** Lianming Wu, Weiwei Xu, Yanzi Hu, Jing Chen

**Affiliations:** a Department of General Surgery, Yuhuan Second People’s Hospital, Zhejiang, China; b Department of Radiology, Yuhuan Second People’s Hospital, Zhejiang, China; c Department of Ultrasound, Taizhou Cancer Hospital, Zhejiang, China.

**Keywords:** colorectal liver metastasis, hepatectomy, meta-analysis, radiofrequency ablation

## Abstract

Colorectal cancer (CRC) is one of the most frequently occurring malignancy tumors. Surgical treatment has made great progress in CRC liver metastasis (CRLM), including radiofrequency ablation (RFA), and hepatectomy. We perform a protocol for systematic review and meta-analysis to compare the efficacy and safety of ultrasound-guided RFA and hepatectomy in treating CRLM.

**Methods:**

This systematic review protocol will be reported in accordance with the Preferred Reporting Items for Systematic Review and Meta-Analyses Protocols (PRISMA-P) 2015 Statement. The protocol has been registered in PROSPERO (CRD42022371561). PubMed, EMBASE, MEDLINE, the Cochrane Library, Chinese National Knowledge Infrastructure, Chinese Biomedical Literature Database, Wanfang Database, ClinicalTrials.gov trials registry, and Chinese Clinical Trial Registry will be searched from January 1980 to December 2022. Only randomized controlled trials will be included. Cochrane systematic evaluation tool is used to assess the risk of bias. The RevMan 5.3 software (Cochrane Collaboration, Oxford, UK) will be applied to conduct the meta-analyses.

**Results:**

The results of this systematic review and meta-analysis will be publicly available and published in a peer-reviewed journal.

**Conclusion:**

This study may provide more convincing evidence to help surgeons make decisions when dealing with CRLM.

## 1. Introduction

Colorectal cancer (CRC) is one of the most frequently occurring malignancy tumors which affects nearly 1 million individuals in the world every year.^[1–3]^ CRC patients may develop liver metastasis, which is the major cause of death. Despite significant advances in diagnostic and therapeutic techniques, the survival rate of colorectal liver metastasis (CRLM) patients remains very low.^[4,5]^ CRLM, as a complex cascade reaction process involving multiple factors and procedures, has complex and diverse molecular mechanisms.

The World Health Organization lists CRC as the third leading cause of cancer and the fourth leading cause of cancer mortality in the United States. Due to the portal venous drainage from the colon, the liver is the most frequent site of metastases. Approximately 50% of patients are diagnosed with synchronous or metachronous colorectal liver metastases (CRLM), and it is the leading cause of death in CRC patients.^[6,7]^ Liver metastases are present in 20% to 50% of patients upon initial diagnosis, and the remaining half of CRC patients will develop liver metastases throughout the course of their disease.^[8]^

Hepatectomy is currently the most reliable; hepatectomized patients can now achieve long-term survival.^[9]^ However, recurrence has been reported in 50% to 80% of hepatectomized patients, with remnant liver recurrence being the most common and representing approximately half of all recurrences.^[10]^ Ultrasound-guided radiofrequency ablation (RFA), as a common minimally invasive treatment modality, has been widely used in clinical practice for the local control of liver tumors, and previous reports have demonstrated that thermal ablation had an advantage over surgical resection in being less invasive for hepatocellular carcinoma^[11,12]^; therefore, it can also be an alternative option for patients with unresectable CRLM. Therefore, we perform a protocol for systematic review and meta-analysis to compare the efficacy and safety of ultrasound-guided RFA and hepatectomy in treating CRLM.

## 2. Methods

### 2.1. Study design

This systematic review protocol will be reported in accordance with the Preferred Reporting Items for Systematic Review and Meta-Analyses Protocols (PRISMA-P) 2015 Statement.^[13]^ The protocol has been registered in PROSPERO (CRD42022371561). Given that the meta-analysis is a secondary research which based on some previously published data, ethical approval is not necessary.

### 2.2. Eligibility criteria

#### 2.2.1. Types of studies.

Randomized controlled trial without restriction on the use of blind methods will be included. Other designs, such as review, case reports, retrospective studies and non-randomized controlled trial will be excluded. There are no restrictions on languages.

#### 2.2.2. Types of participants.

For patients diagnosed with CRLM, there are no restrictions on gender, age, primary tumor grade, liver metastasis site, or number of metastases.

#### 2.2.3. Types of interventions.

Patients in intervention group receive ultrasound-guided RFA and patients in control group receive hepatectomy.

#### 2.2.4. Types of outcomes.

Main outcomes are the occurrence of complication (lung infection, incision infection, hemorrhage from liver section), estimated blood loss, the occurrence of relapse (local recurrence, intra-hepatic recurrence, extra-hepatic recurrence) and overall survival rate. Additional outcomes are length of hospital stays and quality of life.

### 2.3. Search strategy

PubMed, EMBASE, MEDLINE, the Cochrane Library, Chinese National Knowledge Infrastructure, Chinese Biomedical Literature Database, Wanfang Database, ClinicalTrials.gov trials registry, and Chinese Clinical Trial Registry will be searched from January 1980 to December 2022. A combination of subject words and free text words will be applied in the searches. The language is limited to Chinese and English. Table 1 presents the retrieval strategy in PubMed.

**Table 1:** Search strategy used in PubMed database.

Number	Search terms
#1	Colorectal liver metastasis [Ti/Ab]
#2	Neoplasm metastasis [Ti/Ab]
#3	Colorectal neoplasm [Ti/Ab]
#4	Liver metastasis [Ti/Ab]
#5	#1 OR #2 OR #3 OR #4
#6	Hepatic resection [Ti/Ab]
#7	Liver resection [Ti/Ab]
#8	Hepatectomy [Ti/Ab]
#9	#6 OR #7 OR #8
#10	Radiofrequency ablation [Ti/Ab]
#11	Pulsed radiofrequency [Ti/Ab]
#12	Radiofrequencies [Ti/Ab]
#13	#10 OR #11 OR #12
#14	Randomized [Ti/Ab]
#15	Randomly [Ti/Ab]
#16	Blind [Ti/Ab]
#17	#14 OR #15 OR #16
#18	#5 AND #9 AND #13 AND #17

### 2.4. Study selection

**Figure 1.**
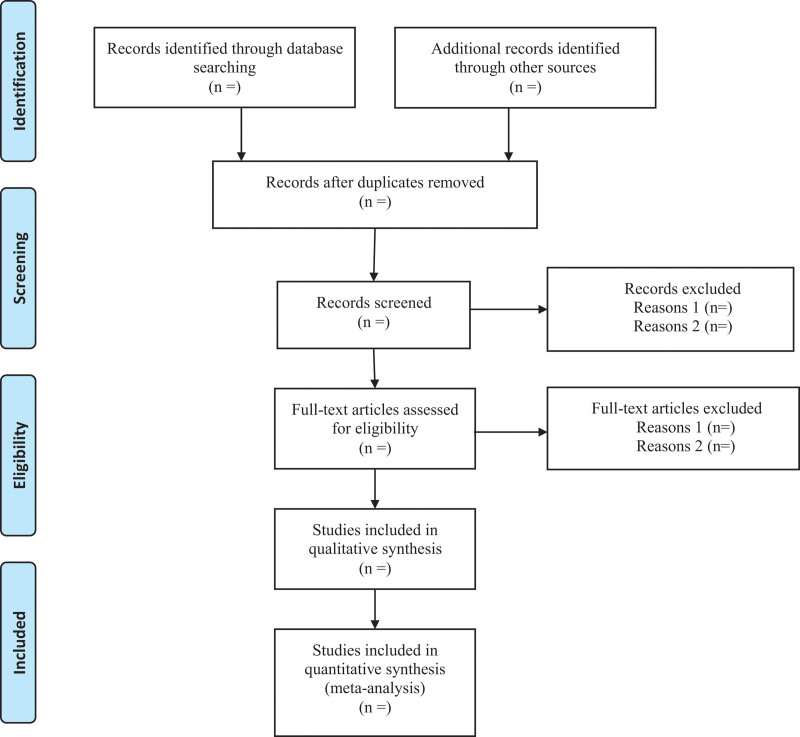
Flow diagram of study selection process.

All retrieved records will be imported into Endnote X9.1 software and the duplicated records will be removed. For studies that have been updated, the older one will be excluded, or can be used as supplementary data in further research. Titles and abstracts will be screened independently by 2 reviewers. Full texts will be obtained for eligible studies and will be screened independently. Discrepancies will be resolved through discussion, or by consulting a third reviewer. The procedures of study selection will be performed in accordance with the Preferred Reporting Items for Systematic reviews and Meta-Analysis flow chart (as shown in Fig. 1).

### 2.5. Data extraction

First, we will extract and record the first author’s name, year of publication, study design, group information, age, gender, dropouts, sample size, duration of intervention and outcome from the studies that meet the inclusion criteria. We will contact the corresponding authors for additional information if necessary. Disagreements will be resolved by discussion by arbiter.

### 2.6. Risk of bias

Two researchers will use the Cochrane systematic evaluation tool to assess the risk of bias in included trials independently.^[14]^ The bias tool embodies 7 components: random sequence generation, allocation concealment, blindness of participants and caregivers, blindness of outcome evaluators, incomplete outcome data, selective outcome reports, and other biases. Each item will be assessed on 3 levels: high risk, low risk, or unclear risk. All disagreements will be resolved by discussion with a third investigator to reach a consensus.

### 2.7. Data analysis

#### 2.7.1. Assessment of heterogeneity.

Chi-squared test (α = 0.1) and *I*^2^ value will be adopted respectively to analyze and determine the heterogeneity of the results of included researches. If *I*^2^ ≤ 50%, it can be deemed that the statistic heterogeneity among trials is negligible, and the fixed effects model will be employed to calculate the effect sizes. Otherwise, the heterogeneity among the trials can be considered significant and random effects model will be used.

#### 2.7.2. Data synthesis.

The RevMan 5.3 software (Cochrane Collaboration, Oxford, UK) will be used to conduct the meta-analyses. The difference of continuous variables in each study will be estimated using mean difference. The standardized mean difference will be used if continuous variables are large or are expressed using different units. The risk ratio and the corresponding 95% confidence interval will be used for the dichotomous variables. Sensitivity analyses will be evaluated by removing studies with high risk of bias or excluding one-by-one. Publication bias was evaluated by funnel plots and Egger test.

### 2.8. Grading quality of evidence

Furthermore, for grading the strength of the evidence for all outcomes from the included data, the Grading of Recommendations Assessment, Development and Evaluation method^[15]^ will be clearly described and documented by 2 independent researchers.

## 3. Discussion

Liver metastasis is the leading cause of cancer-related mortality in patients with CRC. Surgical resection remains the gold standard for treating CRLM and can cure some patients or substantially prolong their survival.^[16,17]^ Recent 5-year survival rates are 30% to 50% as reported.^[6]^ However, most patients are not initially candidates for resection because of disease extent, anatomical location, or comorbidities. In addition, concerns regarding complications and mortality have limited the use of resection. RFA is a widely used minimally invasive modality that provides acceptable local control for small tumors and may be an alternative for treating unresectable CRLM.^[18,19]^ Future comparisons of hepatectomy versus RFA are needed: the first tier of future research studies are studies assessing the comparative effectiveness of RFA versus hepatectomy for palliative treatment of CRLMs; the second tier of future research pertains to studying the patients with RFA vs hepatectomy as the first-line treatment in oncological radical treatment of CRLM.

## Author contributions

Conceptualization: Weiwei Xu.

Methodology: Yanzi Hu.

Writing – original draft: Lianming Wu.

Writing – review & editing: Jing Chen.
